# Retinal Angiomatous Proliferation and Pachychoroid: A Case Report

**DOI:** 10.7759/cureus.104749

**Published:** 2026-03-05

**Authors:** Khalil E Aldaher, Arwa Azmeh, Bassel Wehby, Nawras Alhalabi

**Affiliations:** 1 Department of Ophthalmology, Al Mouwasat University Hospital, Faculty of Medicine, Damascus University, Damascus, SYR

**Keywords:** choroid, pachychoroid, retina, retinal angiomatous proliferation (rap), syria, type 3 macular neovascularization (mnv)

## Abstract

Retinal angiomatous proliferation (RAP), also known as type 3 macular neovascularization (MNV), is a distinct and less common subtype of neovascular age-related macular degeneration (nAMD). This condition is typically associated with a thinner subfoveal choroid than in age-matched control eyes. However, the coexistence of RAP and pachychoroid, which is characterized by increased choroidal thickness and choriocapillaris attenuation, is rarely documented in the literature. In this report, we present the case of a 56-year-old male patient diagnosed with RAP in conjunction with pachychoroid. This case highlights the importance of multimodal imaging techniques, including optical coherence tomography (OCT), fluorescein angiography (FA), and optical coherence tomography angiography (OCT-A), to achieve a comprehensive assessment. Additionally, we emphasize the need for a thorough differential diagnosis to accurately identify and manage such overlapping retinal disorders. We hypothesize that choroidal ischemia secondary to compression of choriocapillaris by choroidal pachyvessels led to outer retinal ischemia and upregulation of vascular endothelial growth factor (VEGF), which caused RAP formation starting at the level of retinal superficial and deep capillary plexus and extended to the outer retina. Thus, our hypothesis supports that choriocapillaris atrophy is a common pathology between these two entities and may explain their coexistence.

## Introduction

RAP or type 3 macular neovascularization is a distinct type of neovascular age-related macular degeneration (nAMD) and is thought to account for 12-15% of newly diagnosed patients with age-related macular degeneration (AMD) [1]. It is described as intra-retinal neovascularization [2], which differentiates it from type 1 (sub-retinal pigment epithelium (RPE)) and type 2 (subretinal) neovascularization. RAP progression has been described using a three-stage classification. Stage I involves intraretinal neovascularization at the level of the deep capillary plexus in the parafoveal region. Stage II involves subretinal neovascularization, and stage III involves extension of neovascularization to the sub-RPE space with retina-choroidal anastomosis [3]. Characteristically, sub-foveal choroidal thickness is significantly thinner in RAP than in age-matched control eyes [4]; in addition, RAP occurs in elderly patients [5], since the age at diagnosis is usually in the age group of 70-90 years, and it is rarely observed under the age of 60 years. In contrast, type 1 neovascularization, including aneurysmal type 1, may be associated with pachychoroid [6]. The onset is usually at a younger age than typical nAMD [7], most commonly in the fifth and sixth decades (40s and 50s). Pachychoroid is characterized by dilated outer choroidal vessels (Haller layer) with attenuation of the overlying choriocapillaris [6]. The coexistence of retinal angiomatous proliferation (RAP) and pachychoroid disease has rarely been described. We report an atypical case of RAP in a 56-year-old man accompanied by pachychoroid.

## Case presentation

**Figure 1:**
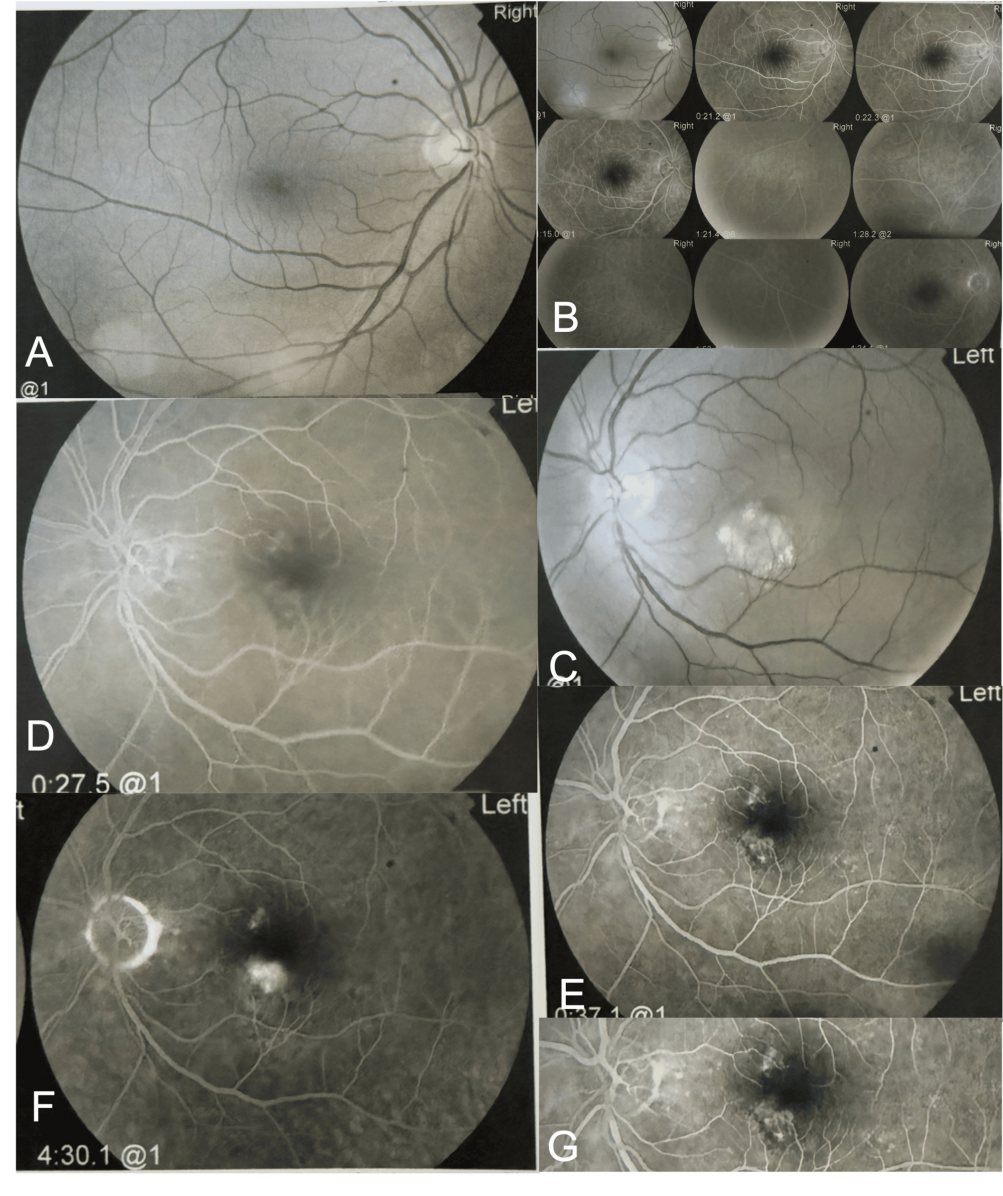
Fluorescein angiography (FA) of both eyes. Red-free photograph of the right eye showed nonspecific pigmentary changes in the central fovea (A) with no abnormalities on FA (B). Red-free photograph of the left eye showed pigmentary changes inferonasal to the fovea (C). The mid venous phase showed two areas of hyperfluorescence: one superonasal and the other inferonasal to the fovea (D). In the late venous phase, the hyperfluorescence of these two areas increased in intensity with the appearance of multiple dots of hyperfluorescence around the macula (E). In the late phase, late leakage was noticed inferonasal to the fovea with no change in the fluorescence of the area superonasal to the fovea (F). The multiple hyperfluorescent dots around the macula faded completely. A diving vessel was noticed in the inferonasal lesion (G).

A 56-year-old male patient was referred to our clinic with acute blurred vision and a central scotoma in his left eye for 10 days. His right eye was asymptomatic. There was no history of diabetes, hypertension, or drug use. Best corrected Snellen visual acuity (BCVA) was 1.0 in the right eye and 0.7 in the left eye. The intraocular pressures were normal in both eyes, and the anterior segments showed no significant abnormalities. Fundus examination of the left eye revealed an abnormal foveal reflex with localized retinal edema and a ring of hard yellow exudates inferonasal to the fovea with no visible drusen. Right fundoscopy was within normal limits. Fundus fluorescein angiography (FA) was normal in the right eye and showed the following in the left eye: The mid venous phase showed two areas of hyperfluorescence, one superonasal and the other inferonasal to the fovea. In the late venous phase, hyperfluorescence in both areas increased in intensity, and multiple punctate hyperfluorescent dots appeared around the macula. In the late phase, late leakage was noticed inferonasal to the fovea with no change in the fluorescence of the area superonasal to the fovea. The punctate hyperfluorescent dots around the macula faded completely. A diving vessel was noticed in the inferonasal lesion (Figure 1).

**Figure 2:**
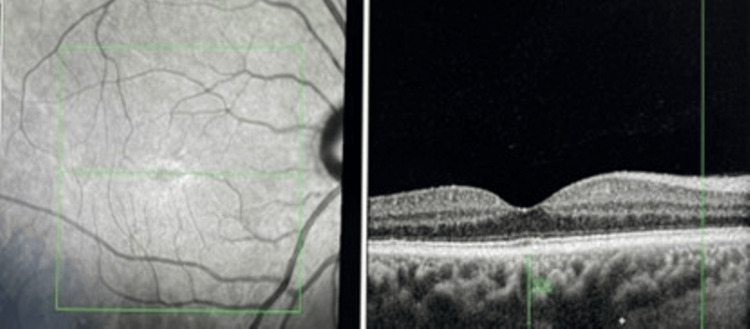
Spectral-domain optical coherence tomography (SD-OCT) across the right fovea showing mild pachychoroid pigment epitheliopathy.

Enhanced depth imaging optical coherence tomography (EDI-OCT) scan across the right fovea showed mild pachychoroid pigment epitheliopathy, and the subfoveal choroid thickness was 404 microns measured from Bruch's membrane to the internal surface of the sclera (Figure 2).

**Figure 3:**
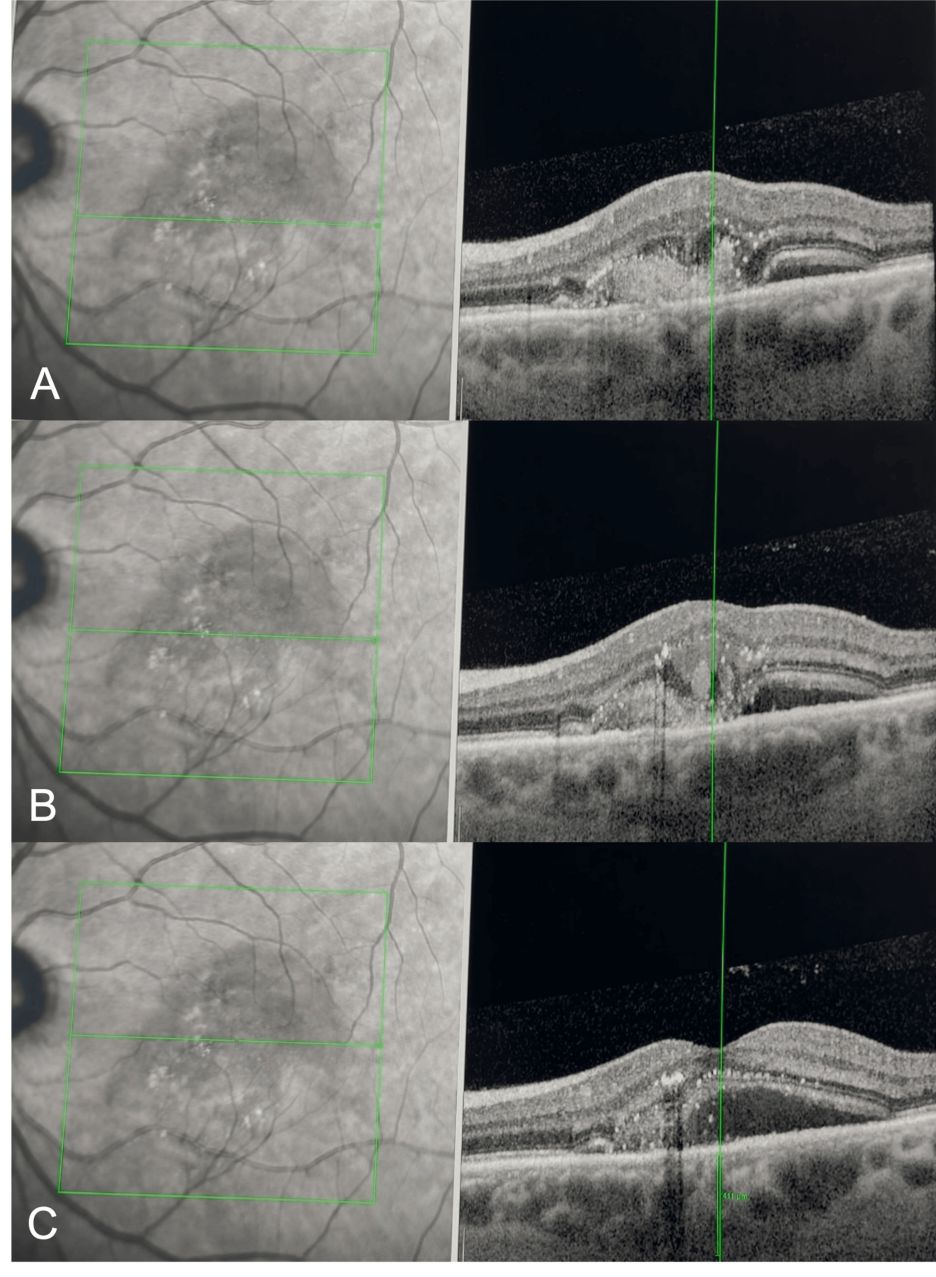
Enhanced depth imaging optical coherence tomography (EDI-OCT) scans across the area inferonasal to fovea of the left eye showing the presence of choroidal thickening (pachychoroid. There is subretinal hyperreflective material with retinal pigment epithelium (RPE) disruption, intra- and sub-retinal fluid (A), two hyper reflective spots at the level of deep capillary plexus, which were thought to be the origin of the retinal angiomatous proliferation (RAP) lesion (B), subfoveal subretinal fluid (SRF) (C).

EDI-OCT scans across the area infero-nasal to fovea of the left eye showed the presence of choroidal thickening (pachychoroid), with subretinal hyperreflective material, RPE disruption, intra- and subretinal fluid, and two hyperreflective spots at the level of deep capillary plexus, which were thought to be the origin of the RAP lesion (Figure 3).

**Figure 4:**
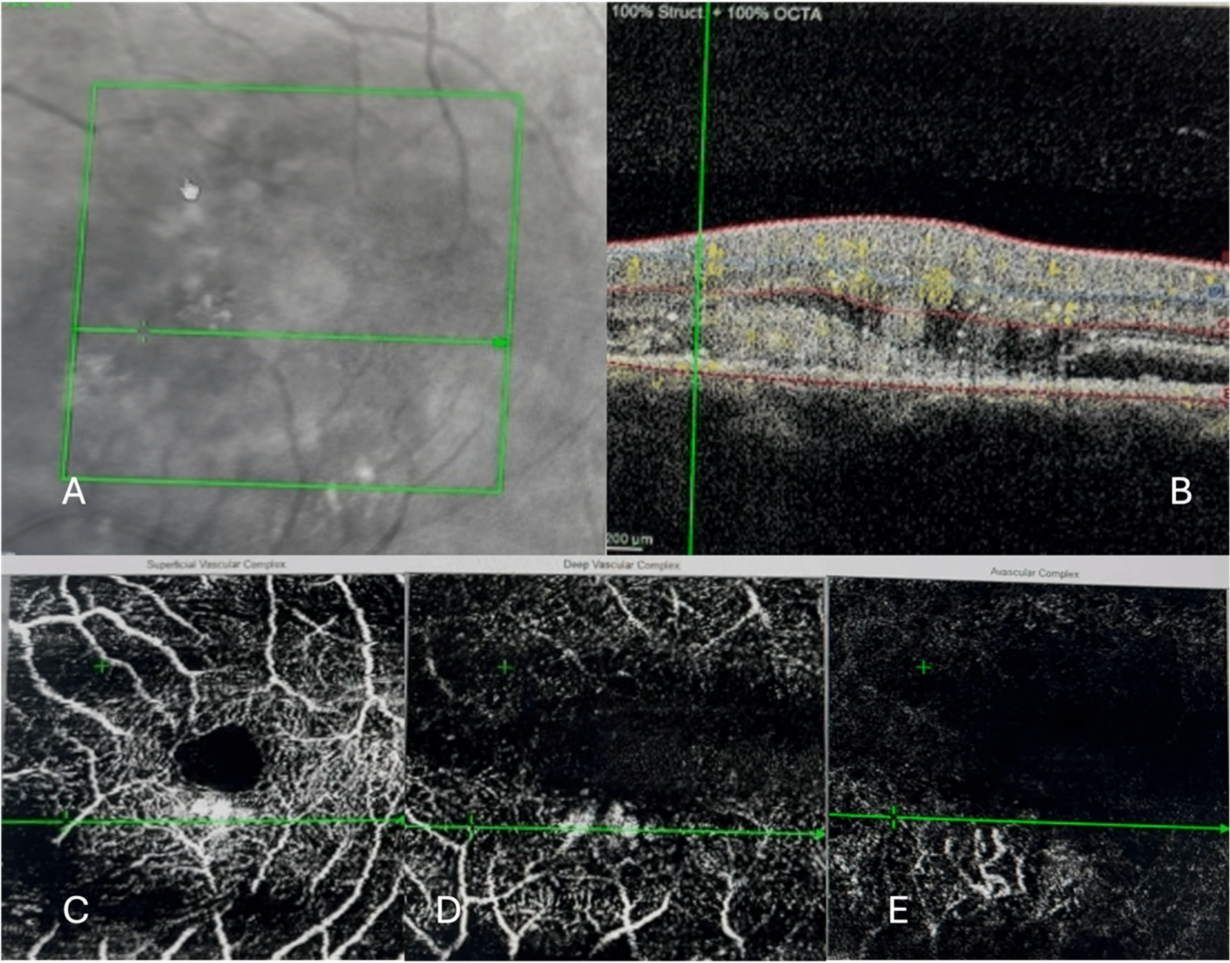
Optical coherence tomography angiography (OCT-A) of the left eye through the area inferonasal to fovea at first presentation. Fundus photograph showing the macular region (A). OCT B-scan through the lesion inferonasal to the fovea (B). OCT-A showing abnormal vascular signals at the level of superficial (C) and deep (D) capillary plexus with type 2 neovascularization at the level of avascular retina (E).

Optical coherence tomography angiography (OCT-A) of the left eye through the area inferonasal to the fovea at first presentation showed parafoveal abnormal vascular signals at the level of superficial and deep capillary plexus, which was thought to be the origin of the RAP, with type 2 neovascularization at the level of avascular retina (Figure 4).

**Figure 5:**
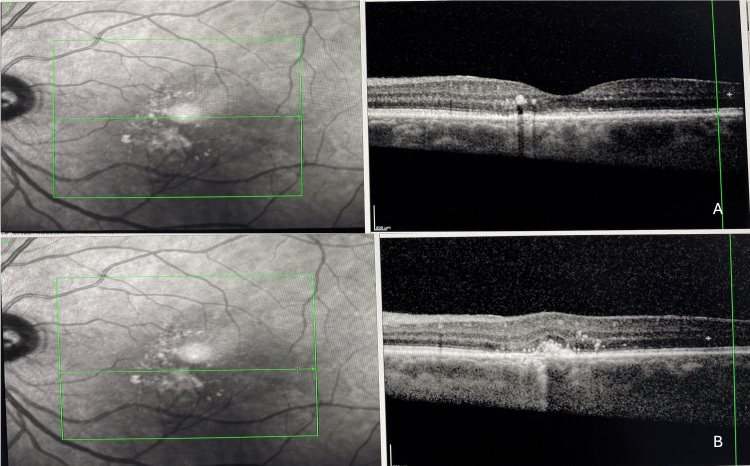
Optical coherence tomography (OCT) of the left eye after two injections one month apart. View through the fovea (A) and inferior to it (B).

**Figure 6:**
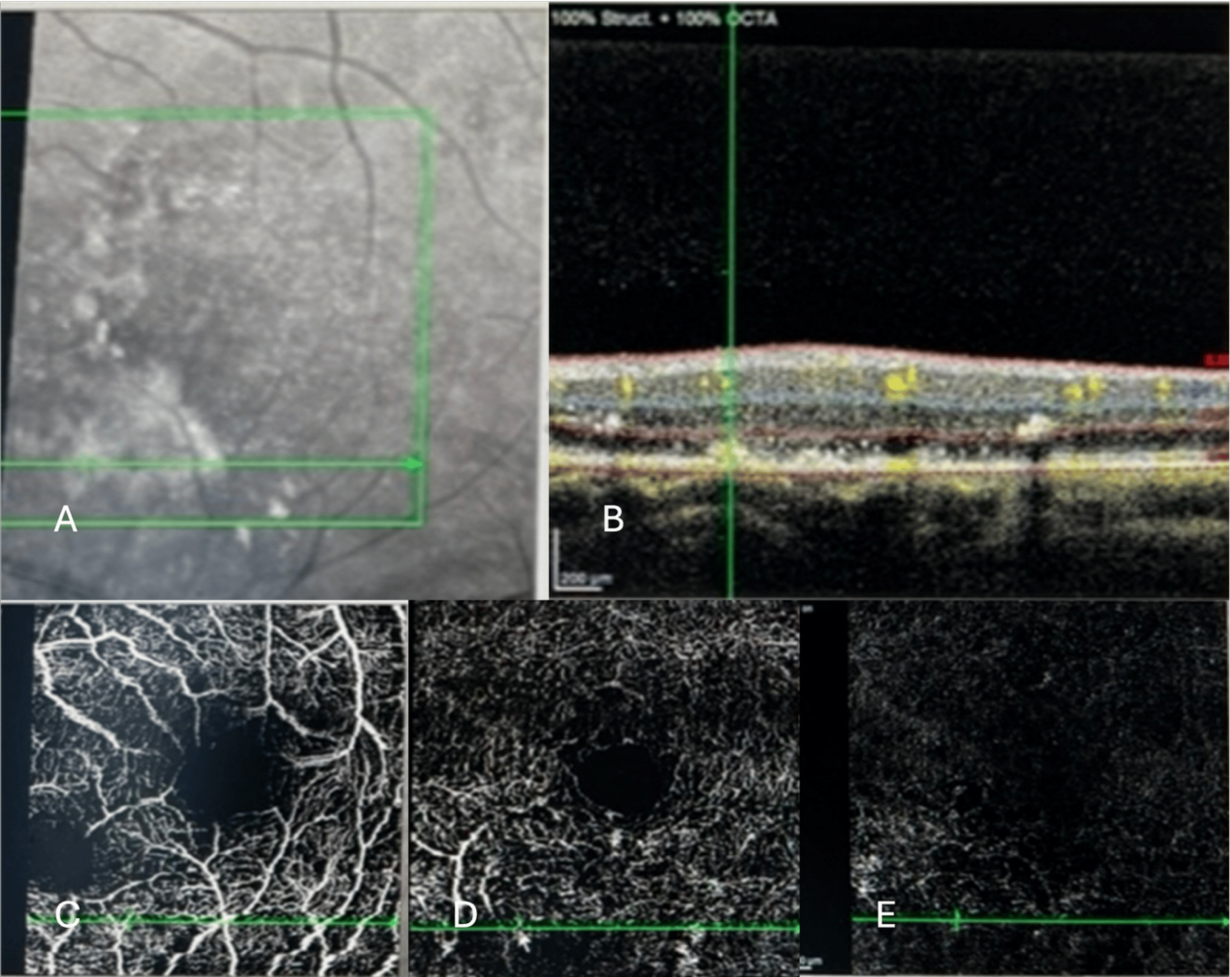
Optical coherence tomography angiography (OCT-A) of the left eye after two injections one month apart. Fundus photograph showing the macular region (A). OCT-B scan through the lesion inferonasal to fovea (B). OCT-A showing the regression of the vascular abnormal signals at the level of the superficial (C) and deep (D) capillary plexus as well as the neovascularization at the level of avascular retina (E) disappeared.

Our differential diagnosis included diabetic macular edema, branch retinal vein occlusion (BRVO), pachychoroid neovascularization, macular telangiectasia type 2, and RAP. As the patient was not diabetic and FA did not show signs of BRVO or telangiectasia type 2, and depending on the findings of EDI-OCT as well as OCT-A, we considered RAP in the eye with pachychoroid to be the most probable diagnosis. Because of the presence of intra- and subretinal fluid, we decided to treat the patient’s left eye with intravitreal bevacizumab (Avastin) with a loading dose of three injections, then as needed. After two injections administered one month apart, BCVA improved to 1.0, the OCT abnormal signs, like sub- and intraretinal fluid and hyperreflective material, and the abnormal OCT-A vascular signals markedly regressed, and the neovascularization at the level of the avascular retina also disappeared (Figures 5, 6).

**Figure 7:**
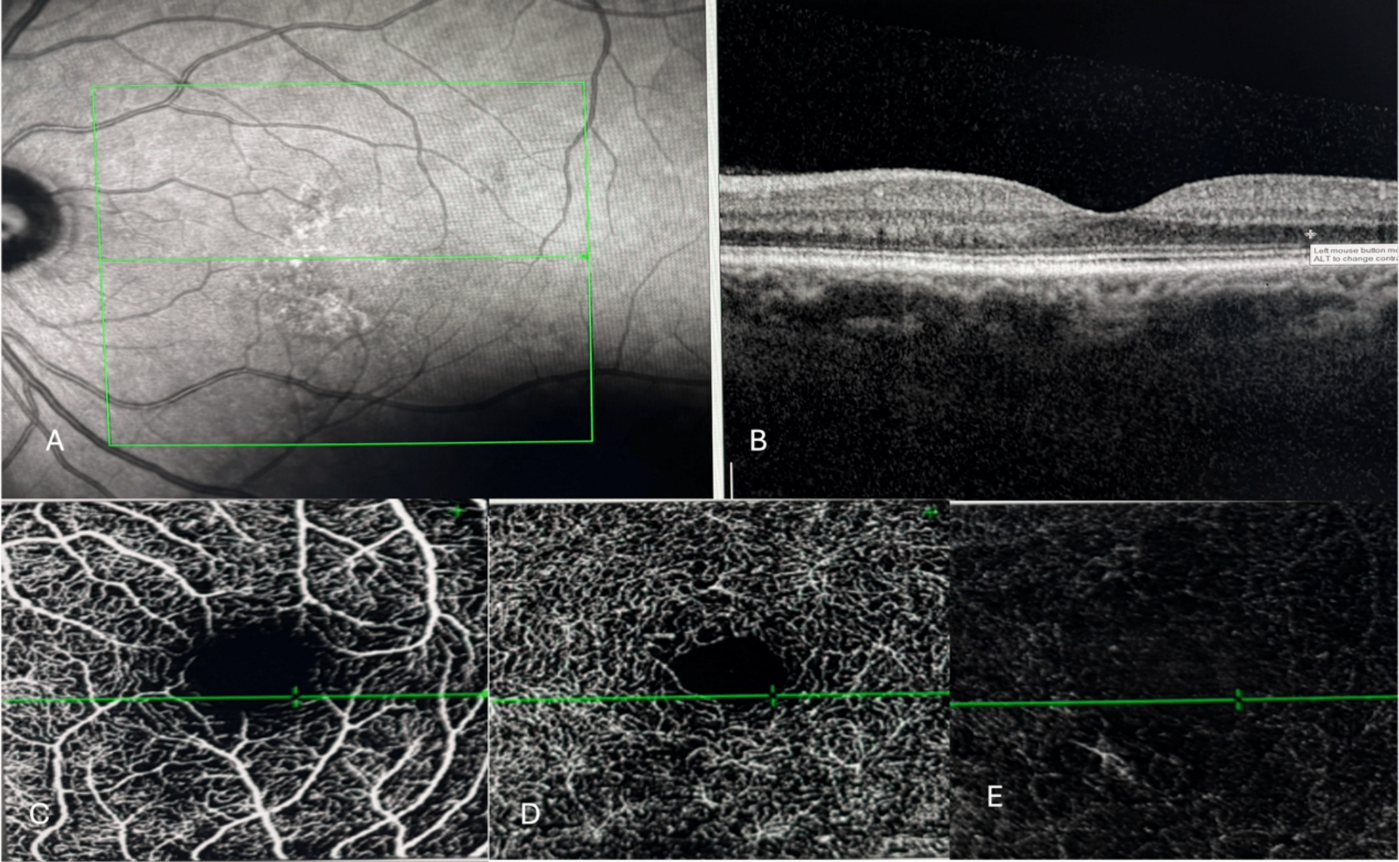
Optical coherence tomography (OCT) and OCT-angiography (OCT-A) of the left eye at the six-month follow-up. Fundus photogragh showing the macular region (A). OCT-B scan through the fovea (B). OCT-A at the level of superfacial (C), deep (D) capillary plexus and avascular retina (E).

The third loading dose injection was done, and during follow-up over the next six months, no recurrence was observed, and the patient's findings remained stable (Figure 7).

## Discussion

The coexistence of RAP and pachychoroid represents a rare but clinically significant finding that may indicate shared pathophysiological mechanisms. RAP is a subtype of neovascular AMD that is typically associated with a thin choroid [8]. It starts with retinal vessels and progresses posteriorly into the subretinal space; eventually, the neovascularization reaches the choroidal circulation and forms retinal-choroidal anastomoses [9]. This is distinct from type 1 MNV in AMD, which originates from the choroid [10] and can erode through the RPE and communicate with the retinal circulation, resulting in a chorioretinal anastomosis [11].

Patients with RAP tend to be older than patients with other forms of neovascular AMD (mean age of 81.7 vs 79, respectively) [12]. The initial lesion is located extrafoveal, presumably because of the lack of capillaries in the foveal avascular zone [8]. Involvement is typically bilateral, with 80% of fellow eyes affected after one year and 100% before three years [13]. Comparing the pathophysiology of RAP and pachychoroid neovasculopathy, we find that both share the presence of choriocapillary attenuation and ischemia. Thickening of RPE and drusen in eyes with RAP hampers the diffusion of vascular endothelial growth factor (VEGF) from RPE to choroid, which disturbs choroidal autoregulation, leading to attenuation of choriocapillaris [14]. Thin choroid reduces the transport of metabolites back to the retina, accentuating the retinal ischemia and leading to RAP [15]. Hypoxia occurs in pachychoroidopathy due to attenuation of the choriocapillary caused by dilated vessels at the level of the Haller layer [16]. This may lead to pachychoroid neovasculopathy.

RAP has rarely been described in association with pachychoroidopathy [17]. A literature review identified only four cases: one eported by Sánchez-Vicente et al. [18], two by Saurabh et al. [17], and one by Song et al. [19]. We report a unilateral extrafoveal case of association between RAP and pachychoroid, in a male patient at an age that is younger for RAP, but consistent with pachychoroidopathy [7]. Our diagnosis was confirmed by FA, EDI-OCT, and OCT-A findings.

RAP demonstrates a good response to anti-VEGF. Eyes with RAP, when treated with intravitreal ranibizumab or bevacizumab, are less likely to have fluid, FA leakage, scar, and subretinal hyperreflective material as compared with all other eyes with nAMD but without RAP, according to the Comparison of Age-Related Macular Degeneration Treatments Trials (CATT) [12]. In the same trial, visual acuity outcomes at one year were better in the RAP group than in the non-RAP group; however, these differences did not persist at year 2. Moreover, eyes with RAP were also found to be more likely to have geographic atrophy compared with eyes without RAP [12]. In the current case, our treatment plan was a loading dose of three injections of bevacizumab, then continuing injections on an as-needed basis. Complete quiescence was noticed after the first injection, with complete disappearance of vascular abnormalities at the level of superficial and deep capillary plexus as well as the avascular retina. This effect continued to be maintained after the second injection, and a visual acuity of 1.0 was achieved. After completing the loading dose, we followed the patient for six months. During this period, no activity was detected until the last visit, and good visual acuity was maintained. This result was in line with the results of Meyerle et al., who noticed complete persistent quiescence within three months of completing the loading dose, with no signs of recurrence [20]. A limitation in our case was the unavailability of indocyanine green angiography in our institution and the need for longer observation to monitor the possibility of recurrence and the incidence of RAP in the other eye.

We hypothesize that choroidal ischemia secondary to compression of choriocapillaris by choroidal pachyvessels in our patient led to outer retinal ischemia and upregulation of VEGF, which caused RAP formation starting at the level of retinal superficial and deep capillary plexus and extended to the outer retina. Thus, our hypothesis supports that choriocapillaris atrophy is a common pathology between these two entities and may explain their coexistence.

## Conclusions

RAP should be considered as one of the various forms of neovascularization associated with pachychoroidopathy. This case highlights the importance of considering RAP in the differential diagnosis of pachychoroid-related disorders. Further studies are needed to improve the understanding of this unique association and to explore the underlying mechanisms that contribute to the coexistence of RAP and pachychoroid. Such research will be crucial in improving diagnostic accuracy and developing targeted therapeutic strategies for patients with these overlapping retinal conditions.
